# Predictors of lack of serological response to syphilis treatment in HIV-infected subjects

**DOI:** 10.7448/IAS.17.4.19654

**Published:** 2014-11-02

**Authors:** Vincenzo Spagnuolo, Andrea Poli, Laura Galli, Massimo Cernuschi, Silvia Nozza, Myriam Maillard, Nicola Gianotti, Hamid Hasson, Simona Bossolasco, Adriano Lazzarin, Antonella Castagna

**Affiliations:** Department of Infectious Diseases, IRCCS San Raffaele Hospital, Milan, Italy

## Abstract

**Introduction:**

The aim of this study was to determine factors associated with lack of serological response (LSR) to treatment of syphilis among HIV-infected subjects.

**Materials and Methods:**

Retrospective, longitudinal study on HIV-infected subjects diagnosed and treated for syphilis and with an assessable serological response between 1 January 2004 and 15 September 2013. LSR was defined as a <4-fold decline of rapid plasma reagin (RPR) titer or a failed reversion to nonreactive (if RPR ≤1:4 at diagnosis) after one year since treatment. Diagnoses of syphilis were staged in early syphilis (primary, secondary and early latent) or late syphilis (tertiary and late latent) according to clinical examination and patient's history. Syphilis was classified in new infections [NI: positive RPR and TPHA (*Treponema pallidum* Haemagglutination assay) titers in subjects without previous history of syphilis] or re-infections [ReI: a ≥4-fold increase of RPR titer in subjects previously successfully treated for syphilis]. Syphilis treatment was prescribed according to CDC guidelines. The crude incidence rates (IRs) of LSR were calculated per 1000-person months of follow-up (PMFU) as the total number of LSR episodes divided by the cumulative time contributed by all subjects (interval time since each syphilis diagnosis and the date of ascertainment of response). Results are described as median (IQR) or frequency (%).

**Results:**

565 diagnoses of syphilis with an assessable serological response in 421 patients; 458 (81%) were early syphilis, 189 (33%) were NI, 376 (67%) were ReI. At first, diagnosis of syphilis median age was 41 (36–47) years, 419 (99.5%) males, 391 (93%) MSM, HIV-infected since 7.7 (3.5–12.9) years, 75 (18%) HCV or HBV co-infected, 56 (13%) with a previous AIDS diagnosis, 82 (19%) antiretroviral treatment naïve, 102 (24%) with HIV-RNA ≥50 cp/mL, CD4+=576 (437–749) cells/mm^3^, nadir CD4+=308 (194–406) cells/mm^3^. LSRs were observed in 70/565 (12.4%) treated syphilis. Incidence of LSR decreased over time [2004–2008 IR=25.1 (17.2–33.1)/1000 PMFU; 2009–2010 IR=21.1 (12.3–29.9)/1000 PMFU; 2011–2013 IR=10.6 (5.1–18.2)/1000 PMFU; Poisson regression: p=0.001]. Results of univariate and multivariate analysis on the risk of LSR are reported in Table 1.

**Conclusions:**

In HIV-infected subjects we observed 12% of LSR to treatment of syphilis. LSR was associated with an older age, late syphilis, lower nadir CD4+ and detectable HIV viral load.

**Table 1:** Univariate and multivariate Cox proportional hazard model for recurrent events on the risk of lack of serological response to treatment of syphilis

	Univariate analysis			Multivariate analysis^a^		
	
Characteristics	HR	95% CI	p	HR	95% CI	p
Age^b^ (per five years older)	1.168	1.083–1.260	<0.001	1.101	1.009–1.201	0.031
Years since HIV infection diagnosis^b^ (per one-year increase)	1.029	1.004–1.054	0.021	1.007	0.958–1.058	0.793
Years of antiretroviral treatment^b^ (per one-year increase)	1.016	0.995–1.037	0.134	1.011	0.954–1.072	0.703
AIDS diagnosis (yes vs no)	1.398	0.923–2.117	0.114	1.078	0.702–1.656	0.732
Nadir CD4+ (per 100 cells/µL higher)	0.800	0.713–0.897	<0.001	0.841	0.742–0.953	0.007
CD4 + b (≤350 vs > 350-cells/µL)	0.940	0.492–1.796	0.852			
HIV-RNA^b^ (≥50 vs < 50 copies/mL)	1.236	0.923–1.855	0.186	1.773	1.143–2.750	0.011
HCV or HBV co-infection (yes vs no)	1.304	0.871–1.953	0.197	0.978	0.658–1.455	0.913
Syphilis stage (late vs early)	1.867	1.216–2.864	0.004	1.962	1.268–3.036	0.003
Type of infection (new infection vs re-infection)	0.705	0.443–1.125	0.141	0.702	0.452–1.091	0.116

a Only covariates with a p < 0.20 at univariate were included in the multivariate analysis

b characteristics at the diagnosis of syphilis. HR = hazard ratio; 95% CI, 95% confidence interval of hazard ratio.

